# Light at night and cause-specific mortality risk in Mainland China: a nationwide observational study

**DOI:** 10.1186/s12916-023-02822-w

**Published:** 2023-03-16

**Authors:** Yao Lu, Peng Yin, Jie Wang, Yiping Yang, Fei Li, Hong Yuan, Shenxin Li, Zheng Long, Maigeng Zhou

**Affiliations:** 1 Clinical Research Center, the Third Xiangya Hospital, Central South University, Changsha, 410013 China; 2 National Center for Chronic and Noncommunicable Disease Control and Prevention, Chinese Center for Disease Control and Prevention, Xicheng District, Beijing, 100050 China; 3 Department of Cardiology, The Third Xiangya Hospital, Central South University, Changsha, 410013 China; 4 Department of Surveying and Remote Sensing Science, School of Geosciences and Info-Physics, Central South University, Changsha, 410083 China

**Keywords:** Light at night, Mortality, Cause-specific mortality, Nationwide study, Time-stratified case-crossover study

## Abstract

**Background:**

While epidemiological studies have found correlations between light at night (LAN) and health effects, none has so far investigated the impacts of LAN on population mortality yet. We aimed to estimate the relative risk for mortality from exposure to LAN in Mainland China.

**Methods:**

This time-stratified case-crossover nationwide study used NPP-VIIRS to obtain daily LAN data of Mainland China between 2015 and 2019. The daily mortality data were obtained from the Disease Surveillance Point System in China. Conditional Poisson regression models were applied to examine the relative risk (RR) for mortality along daily LAN in each county, then meta-analysis was performed to combine the county-specific estimates at the national or regional level.

**Results:**

A total of 579 counties with an average daily LAN of 4.39 (range: 1.02–35.46) were included in the main analysis. The overall RRs per 100 nW/cm^2^/sr increases in daily LAN were 1.08 (95%CI: 1.05–1.11) for all-cause mortality and 1.08 (95%CI: 1.05–1.11) for natural-cause mortality. A positive association between LAN and all natural cause-specific mortality was observed, of which the strongest effect was observed on mortality caused by neuron system disease (RR = 1.32, 95%CI: 1.14–1.52). The results were robust in both younger and old, as well as in males and females. The more pronounced effect of LAN was observed in median LAN-level regions. Combined with an exposure–response curve, our study suggests a non-linear association between LAN and mortality in China.

**Conclusions:**

Our study shows LAN is associated with mortality in China, particularly for neuron system disease-related mortality. These findings have important implications for public health policy establishment to minimize the health consequences of light pollution.

**Supplementary Information:**

The online version contains supplementary material available at 10.1186/s12916-023-02822-w.

## Background

Light at night (LAN) pollution has been a long-established man-made disturbance and a prominent environmental issue, along with the context of urbanization and industrialization. It is estimated more than 80% of the population in the world may currently live under light pollution (> 14 μcd/m^2^, 8% above natural nighttime brightness), and nearly 90% of the population is exposed to light pollution in China [1]. LAN is becoming a globally widespread environmental pollutant and continuing to expand both in spatial extent and intensity without intervention. Besides, the advent and widespread adoption of electric lighting over the past century has profoundly affected the behavior of many individuals; electric lighting in homes, work environments, and public areas has extended daytime activities into the evening, thus increasing the intensity and duration of night-time exposure to light. The number of studies documenting chronic exposure to LAN impacts on human health has grown dramatically in the last decade [2–7]. LAN may represent an emerging health risk factor, and investigation is urgent to clarify the associated health effects, particularly in highly polluted regions. This information is essential for planning suitable public health policies and for public education about the effects of night light pollution.

Exposure to LAN has been linked to a variety of health disorders in people through the circadian disruption mechanism as circadian rhythm is an important regulator of physiology and disease, coordinating the behavior and physiology of all organs for whole-body homeostasis [8, 9]. A circadian rhythm is endogenously generated and can be modulated by external cues. Circadian disruption due to LAN is common in modern life and contributes to a wide range of human diseases, including coronary heart disease [3], diabetes [4], obesity [5], and cancers [6, 7]. The evidence for the effect of LAN is generally restricted to the incidence risk of disease at the individual level, and no studies have evaluated the effects of LAN on mortality or broader categories of specific causes of mortality. Besides, the LAN data of the previous study was limited by the inferences that can be drawn from satellite images (Defence Meteorological Satellite Programme Operational Line Scan) with an insufficient spatial resolution (5 km) [10]. Therefore, an analysis based on a higher-resolution image is needed to explore the effects of LAN on mortality or disease burden.

Here, we used two established nationwide datasets including 579 main Chinese counties to perform a national assessment of the effects of LAN on mortality. We aimed to examine and compare the associations of LAN with daily all-cause and cause-specific mortality at the national and regional levels, as well as to explore the effects of LAN at different LAN intensity levels. Our study provides insight into light pollution that can improve the planning and implementation of public policy aimed at reducing the environmental and mortality burden.

## Methods

### Data collection

This study was based on two national databases on LAN and cause-specific mortality in 579 counties of Mainland China from 2015 to 2019. These counties were selected according to the death registry of China’s Disease Surveillance. To ensure adequate representation at national levels, surveillance points were randomly selected by an iterative method involving multistage stratification that took into account the sociodemographic characteristics of the Chinese population [11]. In brief, the multistage stratification process involves the following steps: first, counties and districts in each province are divided into four strata based on median urbanization index (i.e., high or low urbanization) and median population size (i.e., high or low population size); then, counties and districts in these four strata are further subdivided into two strata based on the median total mortality rate in each of these four strata in each province. The detailed information of this death registry has been previously described [12]. In total, almost all cities at or above the prefecture level were included in the Disease Surveillance Points System, which included 605 districts and counties (equal to the number of administrative districts in China). Of these surveillance points, 579 counties were included because they had LAN information and more than 3 deaths per day. The daily mortality data from 2015 to 2019 were extracted from China’s Disease Surveillance Points system.

Causes of death were coded by the International Classification of Disease 10th (ICD-10): all causes (codes A00–Z99), accident (referred to as “external cause” in the present study; codes S00–Z99), self-harm (codes X60–X84), non-accident (referred to as “natural cause” in the present study; codes A00–R99), nervous system disease (codes G00–G99), digestive system disease (codes K00–K93), urinary system disease (codes N00–N39), renal failure (codes N17–N19), cardiovascular disease (CVD, codes I00–I99), heart failure (codes I50), stroke (codes I60–I69), coronary heart disease (CHD, codes I20–I25), myocardial infarction (MI, codes I21–I23), respiratory system diseases (codes J00–J99), asthma (codes J45–J46), chronic obstructive pulmonary disease (COPD, codes J41–J44), and cancer (codes C00–C97). “Urinary system disease” included renal failure; “cardiovascular disease” included heart failure, stroke, CHD, and MI; and “respiratory disease” included COPD and asthma. Based on tumor location, we then divided “cancer” into subtypes. “Cancers” included skin cancer, lip/oral/pharyngeal cancer, reproductive system cancer (male or female), hematologic cancer, lung cancer, gastrointestinal cancer, and breast cancer (female).

### Measurement of LAN and other environmental risks

Data on daily LAN for each county from China were derived from the Visible Infrared Imaging Radiometer Suite (VIIRS) carried by the Suomi National Polar-Orbiting Partnership (Suomi NPP) satellite (NPP-VIIRS nighttime light data, https://ngdc.noaa.gov) at a spatial resolution of 750 m × 750 m. The data were started in April 2012, with wavelengths covering from 500 to 900 nm. VIIRS observes the LAN of the earth every 24 h, and the transit time of the NPP satellite is 1:30 a.m. at local time. LAN has a specified dynamic range of approximately 7 orders of magnitude from 3 × 10^−9^ to 0.02 W/cm^2^/sr [13, 14]. Therefore, we estimated an objective measure of LAN in units of radiance (nW/cm^2^/sr). The daily composite NPP-VIIRS LAN data from 2015 to 2019 was obtained from the website. Then, we estimated the objective measure of daily LAN for each included county from 2015 to 2019 based on its geocoded address from the NPP-VIIRS. The daily LAN at the county scale uses the tool “Raster Calculator” in ArcGIS 10.2. The collected LAN records are mainly outdoor artificial LAN. Due to inclement weather conditions, LAN data cannot be collected by artificial satellites on some days (Additional file 1: Fig. S1). Our analyses are based on available data.

We also derived daily particular matter 2.5 (PM_2.5_) concentrations from 2015 to 2019 were derived from the China High Air Pollutants (CHAP) dataset (https://weijing-rs.github.io/product.html), with a spatial resolution of 1 × 1 km^2^. The CHAP dataset has been generated from MODIS/Terra + Aqua MAIAC AOD products together with other auxiliary data (e.g., ground-based measurements, satellite remote sensing products, atmospheric reanalysis, and model simulations) using artificial intelligence by considering the spatiotemporal heterogeneity of air pollution [15, 16]. The estimated annual PM_2.5_ concentrations from the dataset are highly correlated with ground-based measurements (*R*^2^ = 0.94) [16]. These daily PM_2.5_ concentrations were assigned to each county based on the longitude and latitude of the address, through the Baidu Web service API of “Geocoder” with Python version 3.8.3 [17]. The daily temperature, dewpoint temperature, and pressure were calculated using Python based on hourly data, which were collected from the ERA5-Land dataset (https://cds.climate.copernicus.eu/) with a spatial resolution of 10 × 10 km. ERA5-Land has been produced by replaying the land component of the ECMWF ERA5 climate reanalysis. The daily humidity was estimated by combing temperature, dewpoint temperature, and pressure. The unit of daily PM_2.5_, daily temperature, and daily humidity were µg/m^3^, °C, and %, respectively.

### Statistical analysis

In this nationwide observational study, we performed a two-stage analysis to calculate the association between daily LAN and daily mortality in China. In stage 1, we first examined the association between daily LAN and daily cause-specific mortality in each county by using a time-stratified case-crossover design, which has been widely used to assess the effects of environmental risk on adverse health events. In this design, all days during which mortality was reported were selected as the case days, while the control days were selected from the same month and year and matched by day of the week to the case days. To account for the relative risk (RR) of cause-specific mortality associated with an increase in daily LAN, this stage analysis applied a conditional Poisson regression model. To control for confounding effects of weather conditions, we also adjusted for daily temperature, daily humidity, and daily PM_2.5_. In stage 2, we used random-effects models to pool the county-specific estimates for all counties or counties in different groups.

To test the reliability of the main results, we did a series of sensitivity analyses. We modified the modeling choices in stage 1: model 1, adjusted by PM_2.5_ and temperature; model 2, adjusted by PM_2.5_ and humidity; and model 3, adjusted by temperature and humidity. To investigate the different lag structures in the model, we performed conditional quasi-Poisson regression to capture the delayed effects of LAN and PM_2.5_ for the lag 0, lag 1, lag 2, lag 3, and lag 0–1 (average of the present and previous day), respectively. We explored the potential overall lag effects of daily LAN and non-linear association with daily mortality using conditional quasi-Poisson regression combined with distributed lag non-linear model. In this model, a natural cubic smooth function with 7 degrees freedom (df) per year was introduced to account for trends in mortality and seasonality. To control potentially non-linear effects of weather conditions, natural spline functions with 6 df for temperature and 3 df for humidity were also introduced into the model.

To further identify the potential effect modifiers, we also conducted a series of stratified analyses. Firstly, to assess whether the association between LAN and mortality differed across subpopulations, we conducted analyses stratified by age (younger means < 65; old means ≥ 65 years) and sex to confirm the robustness of our results. Secondly, all counties in Mainland China were categorized into 3 levels based on a 5-year average per capita gross domestic product (GDP) from 2015 to 2019, which were derived from the yearbook of statistics in China at provincial levels. Above the national 5-year average per GDP (unit of renminbi (RMB): 59,781.8) was regarded as a high economic level and less than 45,000 RMB as a low economic level. Low, median, and high economic level groups include 172, 198, and 209 counties, respectively. We pooled the county-specific estimates for counties in different economic groups. Thirdly, according to tertiles of the 5-year average of daily LAN estimates from 2015 to 2019, we divide all counties into three levels which correspond to low, medium, and high LAN levels, respectively (first tertile means low LAN level: < 2.67 nW/cm^2^/sr; the second tertile means median LAN level 2.67–3.63 nW/cm^2^/sr; the third tertile means high LAN level: > 3.63 nW/cm^2^/sr). Low, median, and high LAN groups include 193 counties, respectively. We also carried out the same analysis for different LAN levels to further identify the effect of LAN. The post hoc comparisons with the Kruskal–Wallis test were performed to make sure whether the effects of LAN were similar in different subgroups.

The analyses were performed with the R software (version 3.4.2, R Foundation for Statistical Computing). Two-tailed *P* values less than 0.05 were considered statistically significant for all statistical tests.

## Results

### Descriptive statistics

**Fig. 1:**
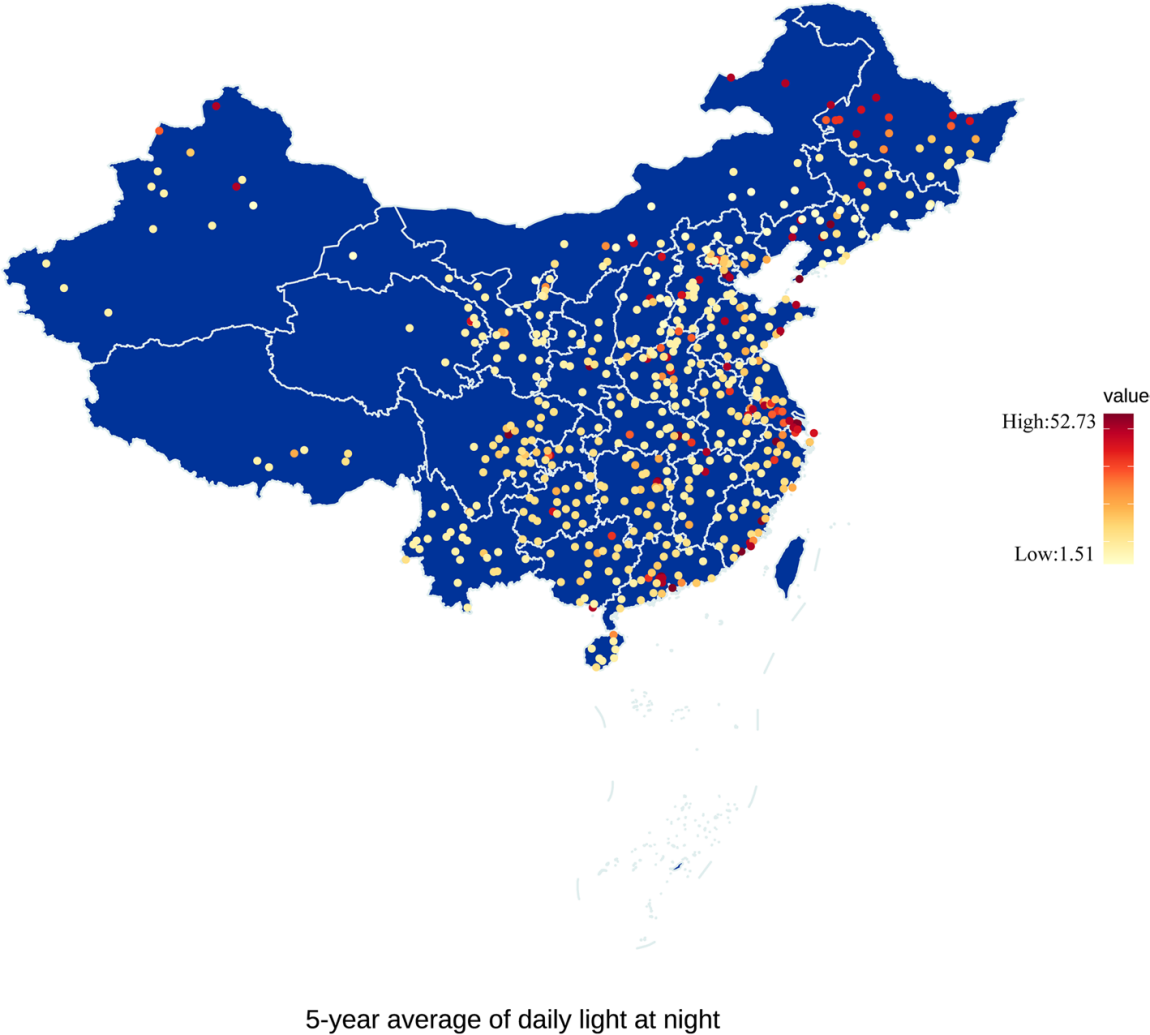
Distribution of the counties with a 5-year average of daily LAN level in Mainland China during the study period (2015–2019). The dot indicates the location of the included county, while the color of the dot indicates the level of LAN, with red denoting a higher LAN level

A total of 579 counties in Mainland China with available data on LAN and mortality were included in the present study during 2015–2019. The spatial patterns of average daily LAN estimations during the study period for each included county in Mainland China were presented in Fig. 1. Compared with most western counties in China, LAN levels were much higher in most eastern counties in China, especially in Shanghai and Beijing. Table 1 provides descriptive statistics on the average number of daily deaths during the study period and the averages of daily LAN and other environmental risks included in the analysis from 2015 to 2019. Large variations in the average LAN intensity of Mainland China were observed with a daily mean LAN of 4.39 nW/cm^2^/sr. Daily average LAN (blue line) and daily number of death (red line) in Mainland China are described in Additional file 1: Fig. S1. The number of all-cause deaths increased along with the daily average LAN during the study period, and similar temporal change trends of two variables were observed. During the study period, we recorded a daily average of 4015 total deaths in 579 counties in Mainland China. There were 291 average daily deaths from external causes and 3724 from natural causes. The number of deaths for different age and sex populations is shown in Additional file 1: Table S1.

**Table 1:** Summary descriptive statistics on the average number of daily deaths and light at night levels in 579 Chinese counties, 2015–2019

Average daily death	Mean (SD)	Range	Median (IQR)
**All-cause death**	4015 (719)	22–7145	3848 (788)
**External**	291 (39)	2–456	287 (42)
**Natural**	3724 (695)	20–6697	3558 (758)
**Neuron system**	51 (13)	2–98	49 (16)
**Digestive system**	92 (17)	1–162	90 (20)
**Cancer**	971 (112)	3–1450	962 (121)
**Urinary system**	45 (11)	0–90	44 (13)
**Cardiovascular**	1819 (398)	5–3469	1725 (448)
**Respiratory system**	466 (137)	5–974	422 (157)
**Average daily LAN (nW/cm**^**2**^**/sr)**			
** National**	4.39 (3.63)	1.02–35.46	2.92 (2.88)
** Low-economic level region**	3.49 (3.42)	0.71–31.59	1.85 (2.91)
** Median-economic level region**	3.67 (3.56)	0.70–30.20	2.15 (2.61)
** High-economic level region**	6.38 (4.04)	1.34–49.90	5.20 (3.33)
** Low LAN level**	2.23 (2.98)	0.30–30.30	0.75 (2.29)
** Median LAN level**	3.15 (4.22)	0.42–36.16	1.10 (3.01)
** High LAN level**	8.15 (3.84)	2.24–44.06	7.17 (3.58)
**Average daily PM**_**2.5**_**(µg/m**^**3**^**)**	35.18 (13.97)	15.13–104.74	31.21 (16.64)
**Average daily temperature (°C)**	16 (9)	− 8–29	18 (17)
**Average daily humidity (%)**	61.20 (7.73)	34.05–79.26	62.12 (11.72)

*LAN* light at night, *PM*_*2.5*_ particular matter 2.5, *SD* standard deviation, *IQR* interquartile range

### Association between LAN and mortality

**Fig. 2:**
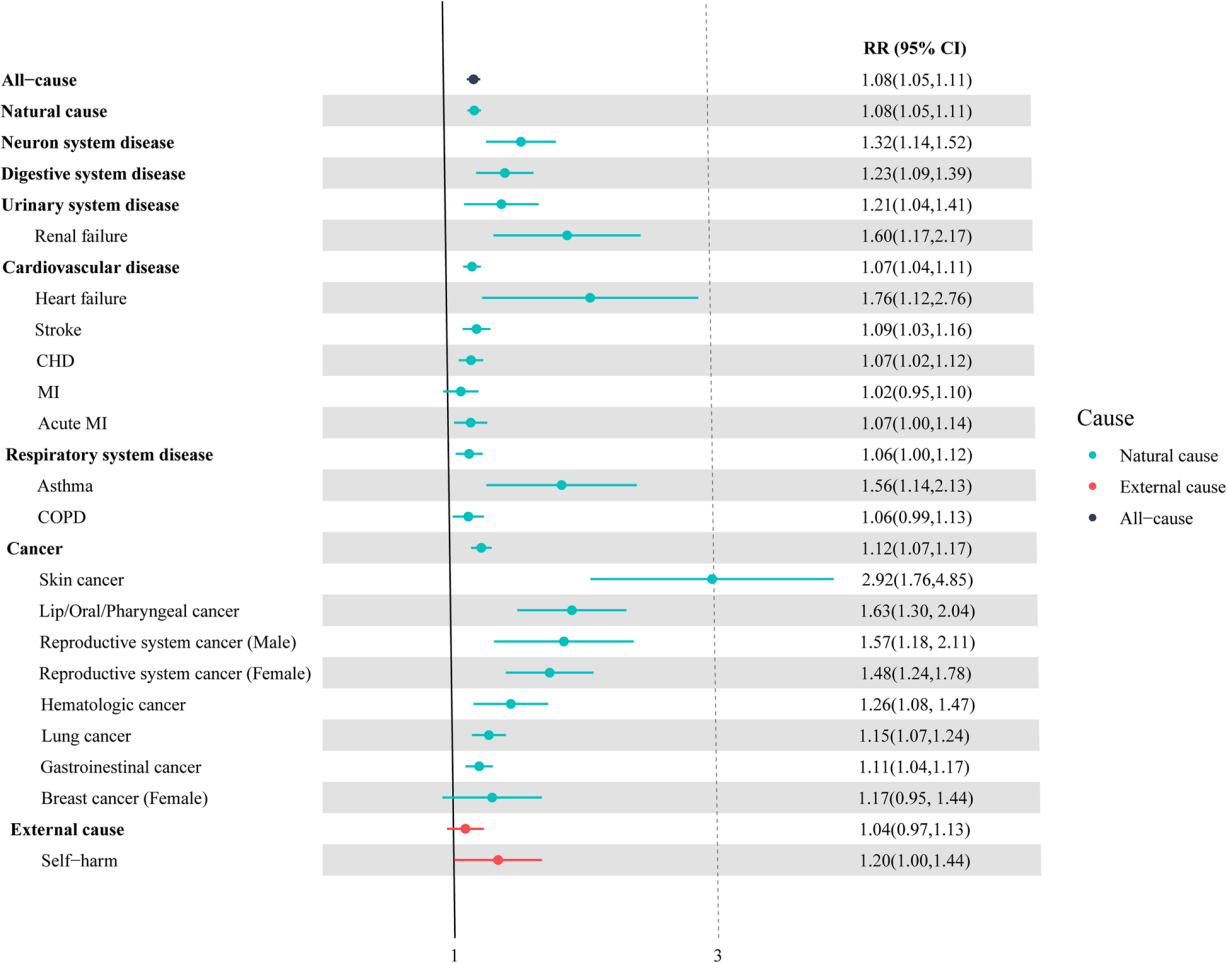
Pooled relative risks of daily specific mortality associated with an increase of 100 nW/cm^2^/sr of daily LAN in 579 Chinese counties, 2015–2019. The results were adjusted by temperature, humidity, and PM_2.5_

At the national level, we estimated that every 100 nW/cm^2^/sr increase in daily LAN was associated with all-cause mortality (RR = 1.08, 95% confidence interval [CI], 1.05–1.11) and natural-cause morality (RR = 1.08, 95%CI = 1.05–1.11), while mortality from external causes was not related to LAN (RR = 1.04, 95%CI = 0.97–1.13) (Fig. 2). In the natural cause-specific mortality analysis, the highest effects of LAN were observed on mortality caused by neuron system disease (RR = 1.32, 95%CI = 1.14–1.52), followed by digestive system disease, urinary system disease, cancer, cardiovascular disease, and respiratory system disease.

In the analysis for specific disease-related mortality, the consistent effects of LAN were also observed in coronary heart disease, stroke, heart failure, asthma, and renal failure. The effects of daily LAN were higher on heart failure, asthma, and renal failure-related death. However, no significant daily LAN-mortality associations were found for mortality caused by MI, COPD, and self-harm (Fig. 2).

In our sensitivity analyses, different adjustments for other environmental risks did not change the results, indicating that our main findings are solid under a series of parameter changes during modeling (Additional file 1: Table S2). Using lag 0, lag 1, lag 2, and lag 3 for LAN and PM_2.5_ did not substantially change the effect of the association between LAN and all-cause and natural cause mortality (Additional file 1: Table S3). In addition, the overall lag effects of LAN, as well as the dose–response relationship between LAN and morality, are shown in Additional file 1: Fig. S2. The effects of LAN on mortality decline as the lag time increases. In Additional file 1: Fig. S2, we have further observed the non-linear association between LAN and morality. The exposure–response curve for LAN did not increase monotonically, with a slight decrease at the high level of LAN exposure, but an upward-sloping at the highest level (Additional file 1: Fig. S2).

### Stratified analysis according to age, sex, and region

When we repeated our analysis within different subgroup populations, our results did not change after stratified by age and sex. Similar to the nationwide estimates, the all-cause and natural cause mortality burden caused by LAN were still robust in various subgroups, while external cause mortality was still statistically insignificant (Table 2). Estimations of respiratory system disease and urinary system disease mortality relative risks were statistically insignificant in younger, and respiratory system diseases were also statistically insignificant in males. Through post hoc comparison, we found the effects of LAN did not differ between the younger group and the old group (*P* = 0.51), nor between males and females (*P* = 0.89).

**Table 2:** Relative risk of daily mortality associated with an increase of 100 nW/cm^2^/sr of daily LAN in 579 Chinese counties, 2015–2019, by subgroup

Death cause	RR (95%CI)			
	**Younger**	**Old**	**Female**	**Male**
**All-cause**	1.05 (1.01, 1.10)	1.09 (1.06, 1.13)	1.07 (1.03, 1.11)	1.09 (1.06, 1.12)
**External**	1.07 (0.97, 1.18)	1.05 (0.95, 1.16)	1.10 (0.96, 1.25)	1.07 (0.98, 1.16)
**Natural**	1.06 (1.02, 1.10)	1.09 (1.06, 1.13)	1.07 (1.03, 1.11)	1.09 (1.06, 1.13)
**Neuron system disease**	1.82 (1.36, 2.45)	1.32 (1.12, 1.56)	1.50 (1.22, 1.85)	1.41 (1.15, 1.73)
**Digestive system disease**	1.39 (1.16, 1.66)	1.24 (1.08, 1.42)	1.21 (1.02, 1.44)	1.33 (1.15, 1.55)
**Urinary system disease**	1.26 (0.95, 1.66)	1.44 (1.19, 1.74)	1.35 (1.06, 1.72)	1.37 (1.12, 1.69)
**Cancer**	1.13 (1.07, 1.18)	1.10 (1.04, 1.17)	1.14 (1.07, 1.21)	1.11 (1.05, 1.17)
**Cardiovascular disease**	1.08 (1.01, 1.15)	1.08 (1.04, 1.12)	1.06 (1.01, 1.11)	1.10 (1.05, 1.15)
**Respiratory system disease**	1.01 (0.86, 1.20)	1.09 (1.03, 1.15)	1.12 (1.02, 1.22)	1.06 (0.98, 1.13)
***P* for post hoc test**	0.51		0.89	

The results adjusted by temperature, humidity, and PM_2.5_

*RR* relative risk, *CI* incidence intervals

When dividing 579 counties into 3 regions by different economical levels, different effects of daily LAN on mortality were not observed according to the results of post hoc analysis (*P* = 0.42). The relationship between daily LAN and mortality was consistent across different levels of economic development (Table 3). These results reflect that the economic level of the regions did not affect the association between daily LAN and mortality. However, when dividing all 579 counties into 3 regions by 5-year average daily LAN level, different effects of daily LAN on mortality were found according to the results of post hoc analysis (*P* = 0.02). Within the median LAN level regions, there is evidence for more significant associations between daily LAN and mortality (Table 3).

**Table 3:** Relative risk of daily mortality associated with an increase of 100 nW/cm^2^/sr of daily LAN in 579 Chinese counties, 2015–2019, by economic development levels and LAN levels

Death cause	RR (95%CI)		
	**High development level**	**Median development level**	**Low development level**
**All-cause**	1.06 (1.03, 1.10)	1.10 (1.04, 1.17)	1.07 (1.02, 1.12)
**External**	1.03 (0.93, 1.13)	1.04 (0.88, 1.23)	1.06 (0.93, 1.22)
**Natural**	1.07 (1.03, 1.10)	1.11 (1.04, 1.18)	1.07 (1.02, 1.12)
**Neuron system disease**	1.27 (1.05, 1.54)	1.61 (1.20, 2.15)	1.61 (1.20, 2.15)
**Digestive system disease**	1.36 (1.12, 1.65)	1.26 (0.99, 1.60)	1.10 (0.91, 1.33)
**Urinary system disease**	1.19 (0.93, 1.52)	1.20 (0.90, 1.61)	1.26 (0.95, 1.67)
**Cancer**	1.07 (1.01, 1.13)	1.19 (1.09, 1.30)	1.13 (0.81, 1.58)
**Cardiovascular disease**	1.07 (1.02, 1.12)	1.09 (1.004, 1.17)	1.05 (0.99, 1.12)
**Respiratory system disease**	1.02 (0.95, 1.09)	1.09 (0.98, 1.21)	1.13 (1.01, 1.27)
***P* for post hoc test**	0.42		
	**High LAN level**	**Median LAN level**	**Low LAN level**
**All-cause**	1.05 (1.01, 1.09)	1.10 (1.05, 1.15)	1.10 (1.02, 1.18)
**External**	0.97 (0.88, 1.07)	1.09 (0.97, 1.23)	1.09 (0.88, 1.35)
**Natural**	1.05 (1.01, 1.09)	1.10 (1.06, 1.15)	1.10 (1.02, 1.19)
**Neuron system disease**	1.22 (0.99, 1.49)	1.37 (1.08, 1.74)	1.68 (1.08, 2.62)
**Digestive system disease**	1.12 (0.93, 1.34)	1.38 (1.16, 1.64)	1.28 (0.90, 1.81)
**Urinary system disease**	1.08 (0.86, 1.37)	1.34 (1.06, 1.69)	1.30 (0.82, 2.07)
**Cancer**	1.07 (1.01, 1.13)	1.11 (1.03, 1.19)	1.27 (1.13, 1.42)
**Cardiovascular disease**	1.04 (0.99, 1.10)	1.11 (1.05, 1.18)	1.06 (0.96, 1.16)
**Respiratory system disease**	1.03 (0.96, 1.11)	1.06 (0.98, 1.16)	1.19 (0.98, 1.43)
***P* for post hoc test**	0.02		

The results adjusted by temperature, humidity, and PM_2.5_

*RR* relative risk, *CI* incidence intervals, *LAN* light at night

## Discussion

To our knowledge, this is the first study to examine the association between daily LAN and the risk of natural-cause mortality as well as external-cause mortality. In this study, exposure to excessive LAN was associated with an increased risk of all-cause mortality. We used nationwide survey data and a dataset of LAN levels in Mainland China, which allowed for a more convincing analysis of the relationship between LAN and mortality. During the last decades, both outdoor LAN and indoor LAN have increased, mainly because of urbanization, industrialization, lights at home turned on during the night, and new sources of exposure such as smartphones. Collectively, our results suggest that exposure to LAN is a novel and important environmental risk factor that cannot be ignored.

LAN exerts profound effects on disease and mortality by directly and indirectly entraining circadian rhythms, melatonin secretion, and sleep deprivation, all of which are crucial for the fitness and survival of species [18]. Given the primordial dominance of the circadian rhythm over physiological processes, it is apparent that the circadian system is crucial for maintaining synchrony between internal physiology, behavior, and the cues deriving from the external environment [18–20]. When the intrinsic circadian clock is disrupted, substantial serious biological disorders will emerge to affect several systems. Habitually exposure to LAN could not only suppress the secretion of melatonin, a hormone released by the pineal gland that regulates sleep–wake cycles, but also causes inflammatory responses and detrimentally affects the immune system [21]. Thus, LAN could lead to a higher risk of mortality by disrupting our intrinsic circadian rhythms and increasing sympathetic tone.

In the cause-specific mortality analysis, the strongest effect of LAN was found in neuron system disease-related mortality. Disruption of the endogenous local circadian rhythm caused by LAN is one of the biologically plausible mechanisms that potentially explain the strongest association between LAN and mortality due to neuron system disease. The central circadian rhythm generator is located in the hypothalamus suprachiasmatic nucleus (SCN), which contains pacemaker neurons that drive rhythm [22]. Indeed, preclinical and clinical studies have already correlated circadian disruption with the accumulation of neurotoxic proteins and neurodegeneration disease [23–25], including Huntington’s disease, Parkinson’s disease, and Alzheimer’s disease. The mechanisms by which LAN exposure modulates neuron function are multifactorial. LAN-induced neuron dysfunction may also be mediated by direct synaptic inputs from the circadian center in the brain without disturbing the circadian rhythm [26]. Evidence from animal studies shows acute LAN displayed increased proinflammation cytokine expression within the hippocampus [27]. Chronic LAN has also been shown to exacerbate LPS-induced proinflammation cytokine expression within the hippocampus [28]. Imaging studies have also shown that light exposure can influence cortical and subcortical networks involved in cognitive processes, both directly and indirectly [29–31]. The latest published epidemiological study provides direct evidence that LAN exposure is associated with a higher risk of mild cognitive impairment in Chinese [32]. Our findings (RR = 1.32, 95%CI = 1.14–1.52, for neuron system disease-related mortality) also further suggest that excessive LAN exposure in China is associated with a significant neuron system disease burden. The impact of chronic LAN exposure on the neuron system needs further investigation to clarify why LAN has the strongest impact on death from neuron system disease.

In 2007, a statement from The World Health Organization classified shift work that disrupted human circadian rhythms as a probable human carcinogen [33]. Recently, epidemiologic and experimental studies also indicated that cancer is associated with circadian rhythms caused by LAN. Clinical studies have demonstrated a potential association between LAN and the incidence risk of thyroid cancer [34], pancreatic cancer [7], and prostate cancer [35], especially breast cancer [6, 35–37]. The latest meta-analysis [37] showed a positive association between exposure to LAN and the incidence risk of breast cancer, particularly in premenopausal women. Our study further proved a close relationship between LAN and cancer mortality, especially skin cancer. In general, there is a bidirectional relationship between the circadian clock and the cell cycle, and the dysregulation of the shared regulatory and coupling connections between the two pathways can be both necessary and sufficient for tumorigenesis [38].

Our study found that LAN exposure also affects cardiovascular mortality, especially heart failure. This result supports previous findings showing that LAN was linked to a higher risk of cardiovascular disease [3, 39]. A previous cohort study based on 58,692 participants aged 65 years and older followed for a median of 11 years in Hong Kong also found outdoor LAN at the residential address was associated with a higher risk of CHD incidence and mortality [3]. Besides, studies of night shift workers, who tend to be exposed to higher levels of LAN, provide similar insights [40, 41]. A meta-analysis [42] of 34 studies reported that night shift work was associated with vascular events including myocardial infarction, stroke, and coronary events. Another latest meta-analysis [43] also discovered the important role of shift work in cardiovascular disease. The effects of LAN and shift work depend on circadian rhythms, which play a key role in the etiology of CVD [44]. In addition to affecting vascular function, disruption of circadian rhythms may secondarily contribute to cardiometabolic disorders through the deregulation of clock function within adipose tissue, liver, and muscle [45].

In the sub-analysis of specific diseases, the results also showed a stronger association between LAN and renal failure. Consistent with our results, a Chinese cross-sectional study reported that night light index (NLI) at a 5-year moving average was significantly associated with an increased risk of chronic kidney disease prevalence [46]. Okuliarova et al. [47] also revealed that chronic exposure to dim LAN disturbed renal immune and redox homeostasis in animal models. In addition to renal failure, LAN also showed a closer association with asthma. A recent study of Chinese college students similarly showed the strongest effect of LAN on asthma [48]. Further studies are needed to evaluate the corresponding renal failure and asthma disease burden attributable to LAN.

This study had several major strengths. First, to our knowledge, it is the first nationwide investigation of the adverse impact on the mortality of LAN. Compared with previous studies confined to individual cities, our study included 579 counties in China, which provided robust evidence with reliable representatives for our findings. Our findings provide an ecological association between LAN and mortality based on data at the county level, which will be quite important for subsequent policy at the national level, to deal with the abuse of LAN. Secondly, compared with the traditional Defense Meteorological Satellite Program’s Operational Linescan System (DMSP-OLS) nighttime light data, the NPP-VIIRS light data at night have a higher spatial resolution, higher data quality, and no over-saturation problem [49]. Higher spatial resolution (0.75 km) is able to differentiate exposure to LAN from other factors that covary across city districts at fine spatial scales. Thirdly, we evaluated the association between LAN and mortality with analyses of a range of detailed causes of death. Our study provided robust evidence of the mortality risk from both natural and external causes. Finally, our analysis accounted for several potential covarying explanatory factors, including atmospheric pollution, temperature, and humidity explicitly.

There were several limitations to consider. First, we used the nighttime light imagery to evaluate LAN at the county level as surrogate data of individuals’ LAN exposure, which may lead to misclassification of LAN exposure. There are possible variations within the same county based on subject residences. However, previous epidemiological studies regarding other environmental risk factors have used the same approaches to estimate exposure levels. Secondly, we cannot adjust for some potential residual confounding effects at individual levels, including indoor light intensity, wearing an eyepatch, and sleep patterns, all of which may interact with LAN. Thirdly, although we have adjusted for atmospheric pollution and climate, we cannot entirely rule out that LAN exposure may be confounded by noise or other unrecognized environmental factors, which may interact with LAN to influence mortality.

## Conclusions

In conclusion, our study provides comprehensive evidence of positive associations between daily LAN and daily mortality caused by all natural causes, especially for neuron system disease-related mortality. By capturing the population exposed to a wide range of LAN levels in China and including objective measures of other environmental risk factors, our study adds to evidence that LAN is having profound impacts on public health, which substantially extent the existing knowledge. The findings on a national level can help improve public health practices to reduce the disease burden associated with LAN and call for policies on strategies for abating them.

## Supplementary Information

**Additional file 1: Table S1.** Summary descriptive statistics on average number of daily deaths in 579 Chinese counties stratified by sex and age, 2015-19. **Table S2.** RR and 95%CI of daily mortality associated with a 100 nanoWatts/cm^2^/sr increase of daily light at night levels for three models. **Table S3.** RR and 95% CI for association between daily mortality and a 100 nanoWatts/cm^2^/sr increase of daily light at night levels for different lag structure. **Fig. S1.** Basic information about average daily light at night (nanoWatts/cm^2^/sr) and daily number of deaths. **Fig. S2.** RRs and 95% CIs of mortality associated with daily light at night level on different lag days during 2015-2019.

## Data Availability

Data may be available upon request.

## Abbreviations

CHAP: China High Air Pollutants
CHD: Coronary heart disease
CI: Confidence interval
COPD: Chronic obstructive pulmonary disease
df: Degrees freedom
DMSP-OLS: Defense Meteorological Satellite Program’s Operational Linescan System
ICD-10: International Classification of Disease-10
LAN: Light at night
MI: Myocardial infarction
NPP: National Polar-Orbiting Partnership
PM2.5: Particular matter 2.5
RMB: Renminbi
RR: Relative risk
VIIRS: Visible Infrared Imaging Radiometer Suite
