# Supplementary material for: Light at night and cause-specific mortality risk in Mainland China: a nationwide observational study
Source: BMC Med. 2023 Mar 16;21:95. doi: 10.1186/s12916-023-02822-w (PMC10022237; doi:10.1186/s12916-023-02822-w)

**Supplementary Materials**

[Table S1. Summary descriptive statistics on average number of daily deaths in 579 Chinese counties stratified by sex and age, 2015-19. 1](#_Toc32634)

[Table S2. RR of daily mortality associated with a 100 nanoWatts/cm](#_Toc21420)^[2](#_Toc21420)^[/sr increase of daily light at night levels for three models. 2](#_Toc21420)

[Table S3. RR and 95% CI for association between daily mortality and a 100 nanoWatts/cm](#_Toc2118)^[2](#_Toc2118)^[/sr increase of daily light at night levels for different lag structure. 3](#_Toc2118)

[Figure S1. Basic information about average daily light at night (nanoWatts/cm](#_Toc26152)^[2](#_Toc26152)^[/sr) and daily number of deaths. 5](#_Toc26152)

[Figure S2. RRs and 95% CIs of mortality associated with daily light at night level on different lag days during 2015-2019. 6](#_Toc18066)

Table S1. Summary descriptive statistics on average number of daily deaths in 579 Chinese counties stratified by sex and age, 2015-19.

|  | **Male** | | | **Female** | | | **Younger** | | | **Old** | | |
| --- | --- | --- | --- | --- | --- | --- | --- | --- | --- | --- | --- | --- |
| **Average daily death** | **Mean (SD)** | **Range** | **Median (IQR)** | **Mean (SD)** | **Range** | **Median (IQR)** | **Mean (SD)** | **Range** | **Median (IQR)** | **Mean (SD)** | **Range** | **Median (IQR)** |
| **All-cause death** | 2337 (398) | 15-4071 | 2246 (430) | 1678 (325) | 7-3074 | 1602 (369) | 1039 (139) | 11-1769 | 1015 (132) | 2920 (590) | 8-5263 | 2782 (657) |
| **External** | 194 (27) | 2-306 | 192 (189) | 98 (17) | 0-172 | 96 (86) | 158 (24) | 1-260 | 156 (29) | 117 (22) | 1-220 | 114 (27) |
| **Natural** | 2144 (385) | 13-3779 | 2054 (421) | 1580 (313) | 7-2918 | 1509 (356) | 882 (125) | 10-1519 | 858 (129) | 2804 (574) | 7-5095 | 2668 (644) |
| **Neuron system** | 26 (8) | 2-56 | 25 (9) | 25 (8) | 0-51 | 24 (9) | 11 (4) | 0-23 | 10 (5) | 38 (11) | 1-75 | 36 (13) |
| **Digestive system** | 60 (12) | 1-103 | 58 (15) | 33 (8) | 0-63 | 32 (10) | 33(8) | 1-62 | 32 (9) | 59 (13) | 0-112 | 57 (15) |
| **Cancer** | 628 (74) | 2-933 | 621 (78) | 344 (42) | 1-538 | 340 (47) | 378 (45) | 3-592 | 374 (44) | 593 (76) | 0-888 | 587 (93) |
| **Urinary system** | 27 (8) | 0-55 | 26 (9) | 19 (6) | 0-42 | 18 (7) | 16 (5) | 0-34 | 15 (7) | 30 (8) | 0-60 | 29 (10) |
| **Cardiovascular** | 988 (213) | 4-1878 | 941 (241) | 832 (189) | 1-1591 | 787 (216) | 333 (61) | 1-618 | 322 (71) | 1485 (342) | 4-2851 | 1405 (391) |
| **Respiratory system** | 271 (78) | 1-529 | 247 (89) | 196 (61) | 4-445 | 177 (71) | 45 (14) | 1-99 | 42 (16) | 418 (124) | 1-868 | 377 (147) |

Table S2. RR of daily mortality associated with a 100 nanoWatts/cm^2^/sr increase of daily light at night levels for three models.

| Death Cause | RR (95%CI) | | |
| --- | --- | --- | --- |
|  | Model 1 | Model 2 | Model 3 |
| All-cause | 1.07 (1.04,1.10) | 1.08 (1.05,1.11) | 1.10 (1.07,1.12) |
| External | 1.04 (0.96,1.12) | 1.05 (0.98,1.13) | 1.08 (1.01,1.15) |
| Natural | 1.08 (1.05,1.11) | 1.08 (1.05,1.11) | 1.10 (1.07,1.12) |
| Neuron system disease | 1.16 (1.01,1.34) | 1.33 (1.15,1.54) | 1.32 (1.18,1.48) |
| Digestive system disease | 1.48 (1.22,1.80) | 1.20 (1.06,1.34) | 1.19 (1.09,1.30) |
| Urinary system disease | 1.02 (0.88,1.19) | 1.22 (1.04,1.42) | 1.16 (1.02,1.31) |
| Cancer | 1.12 (1.07,1.17) | 1.12 (1.07,1.17) | 1.09 (1.06,1.13) |
| Cardiovascular disease | 1.07 (1.03,1.11) | 1.08 (1.04,1.12) | 1.10 (1.07,1.14) |
| Respiratory system disease | 1.05 (1.00,1.11) | 1.06 (1.00,1.12) | 1.08 (1.04,1.13) |

Model1, adjusted by PM2.5 and temperature; Model2, adjusted by PM2.5 and humidity; Model 3, adjusted by temperature and humidity. RR, relative risk; CI, confidence interval.

Table S3. RR and 95% CI for association between daily mortality and a 100 nanoWatts/cm^2^/sr increase of daily light at night levels for different lag structure.

|  | RR (95%CI) | | |
| --- | --- | --- | --- |
|  | All-cause death | Natural death | External death |
| Lag Structure for LAN | | | |
| Lag 0 | 1.027 (1.002, 1.054) | 1.031 (1.005, 1.058) | 0.770 (0.564, 1.051) |
| Lag 1 | 1.036 (1.011, 1.062) | 1.036 (1.011, 1.062) | 1.529 (1.042, 2.243) |
| Lag 2 | 1.031 (1.006, 1.056) | 1.031 (1.007, 1.056) | 1.170 (0.935, 1.463) |
| Lag 3 | 1.054 (1.029, 1.080) | 1.054 (1.028, 1.080) | 1.374 (0.997, 1.893) |
| Lag0-1 | 1.038 (1.008, 1.068) | 1.040 (1.010, 1.071) | 0.957 (0.766, 1.195) |
|  |  |  |  |
| Lag Structure for PM2.5 | | | |
| Lag 0 | 1.027 (1.002，1.054) | 1.036 (1.005,1.058) | 1.010 (0.936,1.090) |
| Lag 1 | 1.026 (1.000，1.052) | 1.030 (1.004,1.056) | 1.008 (0.933,1.089) |
| Lag 2 | 1.023 (0.998，1.049) | 1.027 (1.002,1.054) | 1.010 (0.935,1.090) |
| Lag 3 | 1.023 (0.998，1.049) | 1.027 (1.002,1.054) | 1.008 (0.934,1.088) |
| Lag0-1 | 1.026 (1.000，1.052) | 1.030 (1.004,1.057) | 1.009 (0.935,1.090) |

RR, relative risk; CI, confidence interval. Results adjusted for temperature and humidity, and PM2.5.


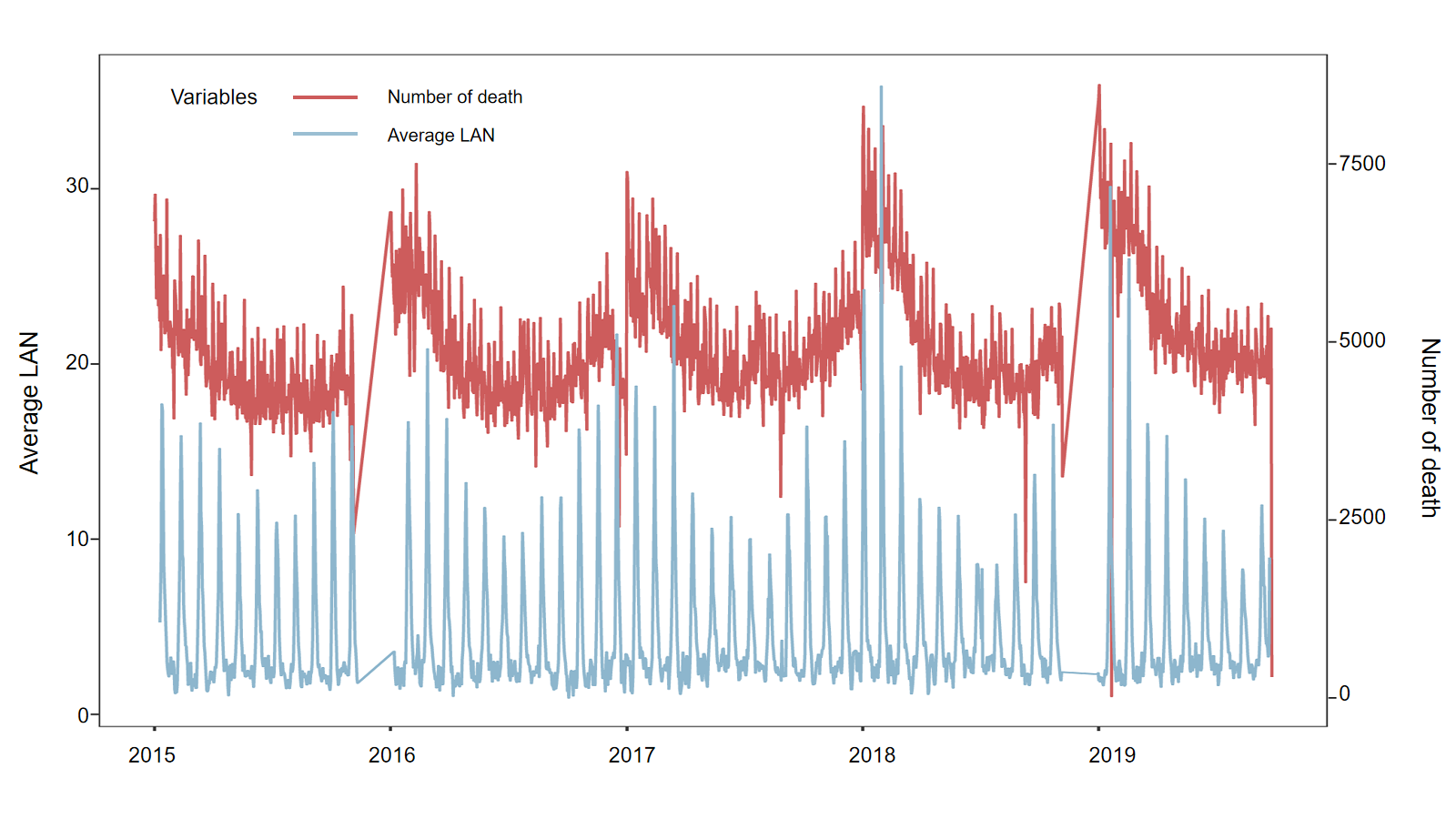
Figure S1. Basic information about average daily light at night (nanoWatts/cm^2^/sr) and daily number of deaths. LAN, light at night.

Figure S2. RRs and 95% CIs of mortality associated with daily light at night level on different lag days during 2015-2019. RR, relative risk; CI, confidence interval; LAN, light at night.


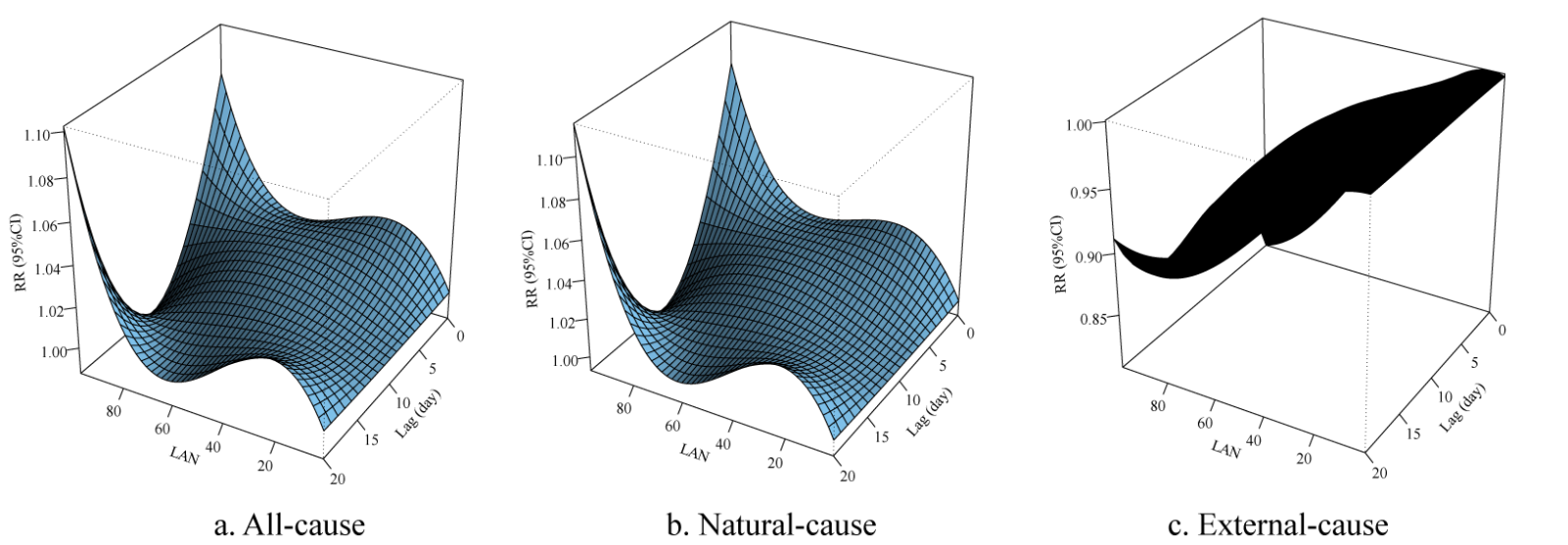

Supplement: Supplementary file 1 — Additional file 1: Table S1. Summary descriptive statistics on average number of daily deaths in 579 Chinese counties stratified by sex and age, 2015-19. Table S2. RR and 95%CI of daily mortality associated with a 100 nanoWatts/cm2/sr increase of daily light at night levels for three models. Table S3. RR and 95% CI for association between daily mortality and a 100 nanoWatts/cm2/sr increase of daily light at night levels for different lag structure. Fig. S1. Basic information about average daily light at night (nanoWatts/cm2/sr) and daily number of deaths. Fig. S2. RRs and 95% CIs of mortality associated with daily light at night level on different lag days during 2015-2019. [file 12916_2023_2822_MOESM1_ESM.docx]
